# Association Between Dipeptidyl Peptidase-4 Inhibitor Use and Cognitive Functions, Brain-Derived Neurotrophic Factor, and Pentraxin-3 Levels in Patients With Type 2 Diabetes

**DOI:** 10.7759/cureus.54440

**Published:** 2024-02-19

**Authors:** Ozan Demirkılıç, İlker Eski, Ece Çiftçi Öztürk, Özge Yasun, Burak Aydın, Can Birkan, Ayşegül Özsoy, Selçuk Şen

**Affiliations:** 1 Division of Clinical Pharmacology, Department of Medical Pharmacology, Istanbul Faculty of Medicine, Istanbul University, Istanbul, TUR; 2 Institute of Graduate Studies in Health Sciences, Istanbul University, Istanbul, TUR; 3 Department of Molecular Oncology, Institute of Health Sciences, Istinye University, Istanbul, TUR; 4 Department of Emergency Medicine, Haseki Training and Research Hospital, Istanbul, TUR; 5 Department of Internal Medicine, Haseki Training and Research Hospital, Istanbul, TUR

**Keywords:** pentraxin-3, bdnf, brain-derived neurotrophic factor, cognitive functions, diabetes, dipeptidyl peptidase-4 inhibitors

## Abstract

Background

Diabetes mellitus is an important risk factor for dementia, Alzheimer’s disease, and other neurodegenerative diseases. Recent findings have made the relationship between the inhibition of the dipeptidyl peptidase-4 (DPP-4) enzyme and cognitive functions an important research topic.

Objective

This study aimed to evaluate the association between DPP-4 inhibitor use and cognitive functions, serum brain-derived neurotrophic factor (BDNF), and pentraxin-3 (PTX-3) levels in patients with type 2 diabetes, compared with the patients who only use metformin treatment.

Design, patients, and methods

A total of 50 patients with type 2 diabetes (hemoglobin A1c levels at ≤%7.5) who were under treatment with metformin±DPP-4 inhibitor (n=25) or only metformin (n=25) were included in this cross-sectional study. Serum BDNF and PTX-3 levels were assessed using an enzyme-linked immunosorbent assay. A standardized mini-mental test (sMMSE) was used to evaluate cognitive functions.

Results

There were no significant differences in the characteristics of the study groups. The mean sMMSE score of the patients receiving DPP-4±metformin treatment was statistically higher when compared with patients receiving only metformin treatment (27.16±1.95 vs. 25.40±3.07; p=0.041). The BDNF levels of the patients receiving DPP-4±metformin treatment were considerably higher than the patients receiving only metformin treatment (394.51±205.66 ng/ml vs. 180.63±297.94 ng/ml; p=0.001). The difference in PTX-3 levels between study groups was not statistically significant (5.47±3.44 vs. 3.79±2.53; p=0.055).

Conclusion

When compared to metformin alone, the use of DPP-4 inhibitors in the treatment of patients with type 2 diabetes was associated with increased serum BDNF levels and improved cognitive functions.

## Introduction

Diabetes mellitus is an important risk factor for dementia, Alzheimer’s disease (AD), and other neurodegenerative diseases [1]. In a meta-analysis, an increased risk of 50% to 100% in AD and 100% to 150% in dementia was reported in patients with diabetes [2]. Therefore, early detection and the treatment management of patients with diabetes who are at risk for neurodegenerative diseases including cognitive impairment, dementia, and AD are crucial. Although there is a direct association between high blood glucose levels and neurodegenerative diseases [3], the underlying mechanisms of cognitive impairment in patients with diabetes still remain unclear.

Glucagon-like peptide-1 (GLP-1) is an incretin hormone mainly secreted from intestinal L-cells, pancreatic α cells, and the central nervous system [4]. The main role of GLP-1 in the treatment of diabetes mellitus is to enhance pancreatic beta-cell proliferation and glucose-dependent insulin secretion, thus lowering blood glucose levels. GLP-1 receptors are G-coupled receptors using cyclic AMP as a second messenger. The expression of GLP-1 receptors in the brains of both rodents and humans has been reported earlier [5]. The binding of GLP-1 and its analogs to the GLP-1 receptor may provide neuroprotection against glutamate-induced injury and oxidative injury, as demonstrated in neuronal cell culture studies [5]. Additionally, a decrease in amyloid-β (Aβ) peptide levels via GLP-1 administration has been reported in preclinical studies [6]. Previous works on this matter pointed out that GLP-1 can cross the blood-brain barrier, and therefore the central effect of GLP-1 may have the potential to protect against neurodegenerative diseases and reduce Aβ protein precursor and Aβ deposition in the brain [7,8].

The endogenous GLP-1 is very rapidly inactivated by the dipeptidyl peptidase-4 (DPP-4) enzyme within a couple of minutes [9]. DPP-4 inhibitors, also known as “gliptins,” are relatively new drugs used in the treatment of diabetes mellitus. They are mostly used in combination with metformin in daily diabetes practice. It is well known that DPP-4 inhibitors improve glucose-induced insulin secretion, reduce glucagon secretion, and decrease hemoglobin A1c (HbA1c) levels and glycemic fluctuations by preventing the inactivation of GLP-1 in the peripheral circulation [10]. In addition to their main antidiabetic effects, it has been demonstrated that the DPP-4 inhibitors reduce Aβ levels, suppress Aβ accumulation, and reduce Aβ-induced hippocampal neuronal cell death by increasing GLP-1 levels [11]. There is also an association between neuroinflammation and cognitive impairment in patients with type 2 diabetes [12]. Previous studies illustrated that DPP-4 inhibitors suppress neuroinflammation by reducing the levels of proinflammatory cytokines [13,14]. Keeping all this information in mind, previous findings have made the relationship between the inhibition of the DPP-4 enzyme and cognitive functions an important research topic.

Brain-derived neurotrophic factor (BDNF) is a member of the neurotrophin family and mainly contributes to the growth and differentiation of neurons and protection against neurotoxicity. BDNF has a remarkable role in the modulation of memory and cognition-related neuroplasticity in the hippocampus. Lower levels of BDNF have been reported in patients with neurodegenerative diseases such as AD and Parkinson’s disease [15]. In our study, we also evaluated the level of serum pentraxin-3 (PTX-3), which is a modulator of the inflammatory process, and increased serum levels of PTX-3 have been found in patients with atherosclerotic cardiovascular disease and a variety of diseases accompanied by inflammation [16]. A standardized mini-mental test (sMMSE), a scale for predicting the severity of cognitive impairment and monitoring the patients, was used to evaluate the cognitive functions.

The present study aimed to evaluate the effect of DPP-4 inhibitors on sMMSE scores, serum BDNF, and PTX-3 levels in patients with type 2 diabetes, compared with patients who only use metformin treatment. Patients who received antidiabetic treatments other than DPP-4 inhibitors and metformin were not included in the study. There was no significant difference in patients’ characteristics between study groups, and we recruited only the patients with diabetes who have HbA1c levels at ≤7.5% to evaluate the effects of DPP-4 inhibitors on study parameters, independent of the blood glucose-lowering effect.

## Materials and methods

A total of 50 patients with type 2 diabetes who were under treatment with metformin±DPP-4 inhibitor (n=25) or only metformin (n=25) for at least three months and presented to the Istanbul Medical Faculty Department of Clinical Pharmacology Polyclinic or Health Sciences University Haseki Traning and Research Hospital Internal Medicine Polyclinic were included in this cross-sectional study. The study was conducted in accordance with the Declaration of Helsinki and approved by the Istanbul Faculty of Medicine Clinical Research Ethics Committee (approval no. 682028, date: 03.01.2022). All enrolled participants gave their written informed consent. Patients who had type 2 diabetes, followed-up outpatients, were speaking and writing in Turkish, and at least five years of education were included. Patients who had type 1 diabetes, were on insulin or antidiabetic treatments other than DPP-4 inhibitors and metformin, had an HbA1c value >7.5%, had a history of cerebrovascular disease, had any disease of the central nervous system, had significant hepatic disease (alanine aminotransferase (ALT)/aspartate aminotransferase (AST) >2 times the upper limit of normal), and had significant renal disease (glomerular filtration rate (GFR) <60 mL/min according to the Cockroft-Gault formula) were excluded. Participants were stratified into two groups: patients receiving DPP-4 inhibitors±metformin (n=25) and patients receiving only metformin treatment as the control group (n=25).

Sociodemographic parameters, including age, gender, and duration of diabetes; presence of hypertension and hyperlipidemia; systolic and diastolic blood pressure levels; and the laboratory test results of the patients from the last one month (if available), including fasting glucose, HbA1c, total cholesterol, low-density lipoprotein (LDL) cholesterol, high-density lipoprotein (HDL) cholesterol, blood urea nitrogen (BUN), creatinine, AST, ALT, and C-reactive protein (CRP), were recorded and evaluated.

Assessments of serum BDNF and PTX-3 levels

Blood serum specimens were collected to measure BDNF and PTX-3 levels using an enzyme-linked immunosorbent assay (ELISA; Human PTX 3/TSG-14 (pentraxin-3) ELISA kit, Elabscience (Catalog # E-EL-H6081, RRID: AB_2936233); RayBio Human BDNF ELISA Kit (Catalog # ELH-BDNF, RRID: AB_2936234)). Blood samples were collected between 08:00 and 10:00 a.m. Venous blood samples were collected into anticoagulant-free tubes. Blood samples were allowed to coagulate at room temperature for 30 minutes and then centrifuged at 3000 rpm for 10 minutes. Aliquots of serum samples were transported into Eppendorf tubes. All samples were stored at -80 °C as required for analysis. ELISA assays were performed according to the manufacturer’s instructions.

Evaluation of cognitive functions

Firstly, all participants were interviewed to exclude dementia in accordance with the Diagnostic and Statistical Manual of Mental Disorders (5th ed.; DSM-5) criteria [17]. A validated sMMSE in Turkish [18] was used to evaluate the cognitive functions. The score of the test was assessed over 30 points.

Statistical analyses

Statistical analyses were executed using SPSS Statistics version 21.0 (IBM Corp. Released 2012. IBM SPSS Statistics for Windows, Version 21.0. Armonk, NY: IBM Corp.). In the first step, the Shapiro-Wilk test was used to determine whether or not the variables fit the normal distribution. For variables with a normal distribution, a t-test and Pearson’s correlation analyses were used. Mann-Whitney U test and Spearman’s rho correlation analyses were used for variables without a normal distribution. The chi-square test was used to examine the categorical variables. A p-value of <0.05 was considered statistically significant. The results were presented as mean±standard deviation (SD) and median (25th-75th percentiles).

## Results

A total of 50 patients (31 female and 19 male, mean age 51.38±8.42 years) were consecutively enrolled in the study. The study participants were stratified into two groups: patients receiving DPP-4 inhibitors±metformin (n=25) and patients receiving only metformin treatment as the control group (n=25). In the DPP-4 inhibitors±metformin group, vildagliptin (n=7), sitagliptin (n=9), and linagliptin (n=9) were “gliptins” used by the participants. There were no significant differences in the characteristics of the study groups (Table 1).

**Table 1:** Characteristics of the study groups BMI: body mass index, DBP: diastolic blood pressure, SBP: systolic blood pressure, SD: standard deviation *All patients with hypertension were on antihypertensive therapy, **All patients with hyperlipidemia were on statin therapy

	DPP-4±metformin (n=25)			Metformin only (n=25)			p-value
Parameter	Mean±SD	Median (25^th^-75^th^ percentiles)	n (%)	Mean±SD	Median (25^th^-75^th ^percentiles)	n (%)	
Age (year)	51.56±9.26	53 (42-59)		51.20±7.68	49 (45.50-56.50)		p=0.922
Duration of diabetes (year)	4.5±3.54	4 (1-7)		2.4±2.32	1 (1-3)		p=0.108
Education (year)	5.48±1.66	5 (5-5)		6±2.40	5 (5-5)		p=0.253
Weight (kg)	85.46±15.44	85 (75.50-90)		84.94±11.44	86 (78.50-94.50)		p=0.895
BMI (kg/m^2^)	32.13±6.35	30.97 (28.14-35.68)		31.87±4.77	30.60 (28.73-34.56)		p=0.942
SBP (mmHg)	129.60±11.36	130 (120-140)		127.60±15.35	120 (120-140)		p=0.416
DBP (mmHg)	82±8.54	80 (80-90)		80.40±7.35	80 (75-90)		p=0.407
Gender (female/male)			17/8 (68%)			14 /11 (56%)	p=0.382
Hypertension (yes/no)*			13/12 (52%)			8/17 (32%)	p=0.152
Hyperlipidemia (yes/no)**			10/15 (40%)			6/19 (24%)	p=0.225

There were no significant differences in fasting blood glucose (128.68±29.74 mg/dl vs. 120.80±19.32 mg/dl, p=0.272) and HbA1c levels (6.59%±0.52% vs. 6.72%±0.57%, p=0.372) between study groups. Additionally, the other laboratory findings, including total cholesterol, LDL cholesterol, HDL cholesterol, triglyceride, BUN, creatinine, ALT, AST, and CRP levels, did not differ between study groups (Table 2).

**Table 2:** Comparison of laboratory findings between the study groups BUN: blood urea nitrogen, HbA1c: hemoglobin A1c, AST: aspartate aminotransferase, ALT: alanine aminotransferase, CK: creatine kinase, HDL: high-density lipoproteins, LDL: low-density lipoproteins, SD: standard deviation

	DPP-4±metformin				Metformin only				p-value
Parameter	n	Mean±SD	Median (25^th^-75^th^ percentiles)		n	Mean±SD	Median (25^th^-75^th ^percentiles)		
Fasting blood glucose (mg/dl)	25	128.68±29.74	131 (103.50-146.50)		25	120.80±19.32	118 (106.50-132.50)		p=0.272
HbA1c (%)	25	6.59±0.52	6.5 (6.25-7)		25	6.72±0.57	6.9 (6.3-7.2)		p=0.372
Total cholesterol (mg/dl)	24	176.38±41.81	171.50 (144.75-201.25)		21	196.43±36.36	188 (172-225.50)		p=0.096
LDL cholesterol (mg/dl)	24	97.75±35.38	97 (74-115-75)		21	114.04±38.73	111 (85-450.50)		p=0.148
HDL cholesterol (mg/dl)	24	49.75±16.66	48 (39.25-62.25)		21	49.48±12.48	48 (40.50-55.50)		p=0.951
Triglyceride (mg/dl)	24	165.25±98.22	129.50 (101.75-195)		22	183.18±107.71	144.50 (107.75-249.75)		p=0.538
BUN (mg/dl)	19	28.88±7.74	28 (24-35)		24	29.39±8.46	29 (22-33)		p=0.840
Creatinine (mg/dl)	19	0.70±0.16	0.68 (0.60-0.78)		25	0.70±0.17	0.80 (0.59-0.89)		p=0.276
ALT (U/L)	25	18.80±9.04	16 (11.50-23)		23	23±7.76	21 (17-30)		p=0.051
AST (U/L)	25	19.92±7.07	18 (14.50-22)		23	20.65±8.52	19 (15-25)		p=0.733
CRP (mg/l)	10	4.96±2.62	6 (2.1-6.58)		21	4.08±2.12	3.8 (2.25-6.25)		p=0.326

Differences in sMMSE scores, serum BDNF, and serum PTX-3 levels between the study groups

The mean sMMSE score of the patients receiving DPP-4±metformin treatment was statistically higher when compared with patients receiving only metformin treatment (27.16±1.95 vs. 25.40±3.07; p=0.041). The serum BDNF levels of the patients receiving DPP-4±metformin treatment were remarkably higher than the patients receiving only metformin treatment (394.51±205.66 ng/ml vs. 180.63±297.94 ng/ml; p=0.001). On the other hand, the serum PTX-3 levels of the patients receiving DPP-4±metformin treatment were slightly higher than the patients receiving only metformin treatment; however, the difference was not statistically significant (5.47±3.44 vs. 3.79±2.53; p=0.055). The comparison of study parameters between study groups is presented in Table 3.

**Table 3:** Comparison of sMMSE scores, serum BDNF, and serum PTX-3 levels between the study groups BDNF: brain-derived neurotrophic factor, PTX-3: pentraxin-3, sMMSE: standardized mini-mental test, SD: standard deviation *p<0.05, **p<0.01

	DPP-4±metformin (n=25)		Metformin only (n=25)			p-value
Parameter	Mean±SD	Median (25^th^-75^th^ percentiles)	Mean±SD		Median (25^th^-75^th ^percentiles)	
sMMSE score	27.16±1.95	27 (25.50-28.50)	25.40±3.07		26 (23-28)	p=0.041*
Serum BDNF levels (ng/ml)	394.51±205.66	471.75 (274.85-540.89)	180.63±297.94		48.56 (16.08-385.72)	p=0.001**
Serum PTX-3 levels (pg/ml)	5.47±3.44	3.50 (1.73-5.28)	3.79±2.53		4.67 (2.72-8.00)	p=0.055

Correlations between study parameters

The statistical correlations between age, sMMSE, serum PTX-3 levels, serum BDNF levels, HbA1c, and fasting blood glucose levels were evaluated, and the results are presented in Table 4. The sMMSE score was inversely correlated with age (r=-0.325, p=0.021) and positively correlated with serum BDNF levels (r=0.316, p=0.025). Serum BDNF levels were negatively correlated with HbA1c levels (r=-0.354, p=0.012). On the other hand, there was a significant positive correlation between serum BDNF and PTX-3 levels (r=0.440, p=0.001). The inverse correlation between HbA1c levels and the sMMSE score did not reach a significant level (r=-0.262, p=0.066). As an expected result, there was a strong correlation between HbA1c levels and fasting blood glucose levels (r=0.528, p<0.001).

**Table 4:** Correlations between the study parameters BDNF: brain-derived neurotrophic factor, FBG: fasting blood glucose, HbA1c: hemoglobin A1c, PTX-3: pentraxin-3, sMMSE: standardized mini-mental test *p<0.05, **p<0.01

		Age	sMMSE	PTX-3	BDNF	HbA1C	FBG
Age	Correlation coefficient		-0.325*	0.068	0.135	-0.044	0.044
	p-value		0.021	0.638	0.351	0.763	0.761
sMMSE	Correlation coefficient	-0.325*		0.103	0.316*	-0.262	0.046
	p-value	0.021		0.478	0.025	0.066	0.750
PTX-3	Correlation coefficient	0.068	0.103		0.440**	-0.273	0.060
	p-value	0.638	0.478		0.001	0.055	0.681
BDNF	Correlation coefficient	0.135	0.316*	0.440**		-0.354*	-0.004
	p-value	0.351	0.025	0.001		0.012	0.979
HbA1C	Correlation coefficient	-0.044	-0.262	-0.273	-0.354*		0.528**
	p-value	0.763	0.066	0.055	0.012		<0.001
FBG	Correlation coefficient	0.044	0.046	0.060	-0.004	0.528**	
	p-value	0.761	0.750	0.681	0.979	<0.001	

## Discussion

Diabetes mellitus has a remarkable impact on the central nervous system and is counted as an independent risk factor for dementia, AD, and other neurodegenerative diseases [1]. It has been highlighted in the existing literature that DPP-4 inhibitors may improve learning and memory by enhancing GLP-1 levels [19]. In a study conducted on the data of 11,612 patients with type 2 diabetes in Taiwan, it was concluded that the use of DPP-4 inhibitors with a class effect reduced the risk of all-cause dementia (hazard ratio (HR): 0.798; 95% confidence interval (CI): 0.681-0.883; p<0.001), particularly vascular dementia (HR: 0.575; 95% CI: 0.404-0.681; p<0.001), but not in AD (HR: 0.891; 95% CI: 0.712-1.265; p=0.297) when compared to patients not using DPP-4 inhibitors (p<0.001) [20]. Previous findings in this field make research on the effect of the DPP-4 enzyme and its inhibition on cognitive functions an important research topic.

The sMMSE has been a very commonly used screening test to evaluate cognitive functions worldwide for many years. In our study, patients using DPP-4 inhibitors had significantly higher sMMSE scores than patients using only metformin treatment (p=0.041). In a study conducted on retrospective longitudinal data of 240 older type 2 diabetic patients (drug-naive at baseline) affected by mild cognitive impairment and treated with DPP-4 inhibitor plus metformin (n=120) or sulfonylurea plus metformin (n=120) for two years, it was reported that the DPP-4 administration has a protective role against worsening in cognitive functions [21]. In another study conducted on 253 patients with type 2 diabetes, the patients were stratified into two groups: patients receiving sitagliptin (a DPP-4 inhibitor) + metformin and/or insulin treatment and patients receiving only metformin and/or insulin treatment. The 205 patients (52 patients with AD) who completed the study (without any significant differences in baseline characteristics of study groups) were re-assessed after six months. When compared with the baseline values, a significant increase in sMMSE scores (p=0.034) in the sitagliptin group has been reported; on the other hand, in the subgroup analysis of patients without AD, it has been demonstrated that the patients receiving only sitagliptin or insulin achieved higher sMMSE scores when compared to the patients receiving metformin (p=0.024) [22]. In this sense, the higher mean sMMSE score of the patients receiving DDP-4 inhibitors in our study was compatible with the existing findings.

In a study conducted on a total of 420 participants (208 patients with type 2 diabetes and 212 controls), in addition to the lower cognitive test scores, lower levels of BDNF (p<0.001) were reported in patients with type 2 diabetes when compared to controls [23]. Additionally, in another study conducted on the elderly population with normal glucose tolerance, it was shown that there was an inverse correlation between DDP-4 activity and BDNF levels (r=-0.456, p<0.001) [24]. In our study, there was a remarkable difference in serum BDNF levels between patients using DPP-4±metformin treatment and patients using only metformin treatment (394.51±205.66 ng/ml vs. 180.63±297.94 ng/ml; p=0.001). There was also a significant correlation between sMMSE scores and BDNF levels (r=0.315, p=0.025). In addition to the slightly higher mean sMMSE score, the considerably higher BDNF levels of the patients on DPP-4 inhibitor treatment suggest that the DPP-4 inhibitors may have protective effects against cognitive impairment. In consideration of the lack of significant differences in sociodemographic characteristics and laboratory parameters (including HbA1c levels and fasting blood glucose levels) between study groups, these results of the present study can be interpreted as indicating that the protective effects of DPP-4 inhibitors against cognitive impairment may be independent of their glucose-lowering effects. Although an inverse correlation between DPP-4 activity and BDNF levels was reported in the elderly population with normal glucose tolerance [24], to the best of our knowledge, our work is the first study demonstrating higher serum BDNF levels in patients using DPP-4 inhibitors when compared with patients using only metformin treatment.

In addition to Aβ accumulation, previous studies illustrated an increase in levels of proinflammatory cytokines such as IL-1β, IL-6, and TNF-α in patients with type 2 diabetes. Impairment in cognitive functions due to an increased inflammatory response poses a risk for neurodegenerative diseases such as dementia and AD [25]. Therefore, the association of type 2 diabetes with both cognitive functions and inflammation implies an important research area. PTX-3 is counted as an acute-phase biomarker and modulator of inflammation and partially shows functional similarity to CRP. In our study, the serum PTX-3 levels of the patients receiving DPP-4±metformin treatment were slightly higher than the patients receiving only metformin treatment. However, the difference was not statistically significant (5.47±3.44 vs. 3.79±2.53; p=0.055). In a study conducted on 270 patients with type 2 diabetes in Japan, it was reported that three months of sitagliptin treatment resulted in a decrease in PTX-3 levels compared to baseline values (1.88 (0.78)-1.65 (0.63) ng/ml; p=0.0038) [26]. The possible explanation of the different results of PTX-3 levels in our study can be related to the study design. Our work was a cross-sectional study with a relatively small sample size, and there was no comparison between before and after the treatment. On the other hand, a linear statistically significant correlation was found between PTX-3 and BDNF levels in our study (r=0.440, p=0.001). In a different preclinical study, it was demonstrated that recombinant PTX-3 administration after traumatic brain injury in mice provided activation of A2 astrocytes and improved neuronal survival and neurogenesis [27]. Taking into account all these different and controversial findings, the relationship of PTX-3 levels with cognitive functions and BDNF levels requires a thorough investigation in more detail because it was thought that PTX-3 may have regulatory effects on inflammatory pathways at many points apart from acute inflammation. For example, in another evaluation, it was stated that PTX-3 deficiency may be associated with increased inflammation and subsequently may cause cardiac damage and atherosclerosis. Attention has been drawn to the hypothesis that PTX-3 can modulate inflammation, and increased PTX-3 levels in patients with cardiovascular risk may reflect a physiologically protective mechanism associated with the immunoinflammatory response observed in various cardiovascular diseases [28]. It is a fact that further clinical studies in larger patient populations are required to reach a more definitive conclusion about the impact of sensitive inflammatory biomarkers such as PTX-3.

Although patients with uncontrolled diabetes (HbA1c levels above 7.5%) were not included in our study and it was conducted on a relatively small sample size, a statistically significant inverse correlation was found between serum BDNF level and HbA1c (r=-0.354, p=0.012), which supports the existing literature. This significant correlation between HbA1c (which is an important indicator of long-term glucose regulation) and BDNF levels shows the impact of the deterioration in glucose regulation on worsening cognitive functions.

Limitations

The present work was conducted in real-world conditions that allow us to evaluate the research topic in daily practice. On the other hand, there are some limitations to our study. Our work was a cross-sectional study. It was not possible to evaluate the change in study parameters before and after the treatment. Secondly, this study was conducted on a relatively small dataset. The other limitation of the study was the lack of information related to possible confounding factors such as smoking, alcohol intake, and physical activities. Lastly, except for FBG and HbA1c, the results of some routine laboratory measurements such as lipid profile, ALT, AST, BUN, creatinine, and CRP were not available for all participants. Nevertheless, the present work can be considered a pilot study, and the significant findings may be useful for designing further larger studies.

## Conclusions

The use of DPP-4 inhibitors in the treatment of patients with type 2 diabetes was associated with increased serum BDNF levels and improved cognitive functions. In line with the literature, these findings suggest a potential protective effect of DPP-4 inhibitors on cognitive impairment in patients with type 2 diabetes. However, larger prospective studies with different designs are required to demonstrate the level of evidence for this potential protective effect and to explain the relationship of sensitive inflammation biomarkers such as PTX-3 with cognitive processes. The relevance of the association of DPP-4 inhibitors with increased serum BDNF levels should be evaluated in future studies.
